# Tele-care intervention performed by parents involving specific task- environment- participation (STEP protocol) for infants at risk for developmental delay: protocol of randomized controlled clinical trial

**DOI:** 10.1186/s12887-022-03126-3

**Published:** 2022-01-20

**Authors:** Camila Resende Gâmbaro Lima, Adriana Neves dos Santos, Mariana Martins dos Santos, Catherine Morgan, Nelci Adriana Cicuto Ferreira Rocha

**Affiliations:** 1 Department of Physiotherapy, Neuropediatrics Section, Federal University of São Carlos, Rod. Washington Luis, km 235, São Carlos, SP 13565-905 Brazil; 2 Department of Health Science, Universidade Federal de Santa Catarina, Rod. Governador Jorge Lacerda, n° 3201 - Km 35, 4, Araranguá, SC 88905-355 Brazil; 3 School of Medicine, Paediatrics and Child Health, Sydney, New South Wales Australia

**Keywords:** Intervention, Home intervention, High-risk infants, Tele-care

## Abstract

**Background:**

With the implementation of social distancing due to the Covid-19 pandemic, many at-risk infants are without therapy. An alternative mode of therapy in this situation is tele-care, a therapy in which assessments and interventions are carried out online, in the home environment. We describe a tele-care protocol involving parent delivered task and context specific movement training, participation and environmental adaptation for infants at risk for developmental delay.

**Methods:**

Randomized controlled trial. Infants at risk, with 3 to 9 months corrected age, will be included, and randomized into two groups: control group (conventional guidelines) and experimental group (task, environment and participation in context-specific home program). Infants will be assessed for motor capacity (Infant Motor Profile and Alberta Infant Motor Scale); participation (Young Children’s Participation and Environment Measure) and environment factors (Parent-Child Early Relational Assessment; Affordances in the Home Environment for Motor Development). The intervention period will be 10 weeks, and evaluations will be carried out before and after that period. All the assessment and intervention procedures will be carried out online, with instructions to parents for home therapy. The statistical analysis will be guided according to the distribution of the data, and a significance level of 5% will be adopted. All ethical approvals were obtained by the Ethics Committee of the University of São Carlos (Case number 31256620.5.0000.5504). The protocol will follow the SPIRIT statement. Findings will be disseminated in peer-reviewed publications and presented at national and international conferences.

**Discussion:**

The results of this study will describe the effectiveness of a home intervention, focusing on specific activities, participation and environmental changes. These results will support the implementation of a remote protocol, with lower financial costs and focused on the particularities of the family. This type of care model can possibly help public policies to ensure equal access to evidence-based quality healthcare.

**Trial registration:**

Brazilian Clinical Trials Registry: RBR8xrzjs, registered September 1, 2020.

**Supplementary Information:**

The online version contains supplementary material available at 10.1186/s12887-022-03126-3.

## Background

Functioning refers to the dynamic and positive interaction between the components of body structure and function, activity and participation, under the influence of environmental and personal factors [1]. Currently, an example of the impact of environment factors on the quality of life is the social barrier caused by COVID-19 pandemic. The high number of people infected by COVID-19 has led to an overload of the health care system, even in high-income countries [2–5].

Social isolation has been the main strategy to delay the spread of the virus [6, 7]. Although social distancing has an essential role in the prevention of the COVID-19 spread, studies have shown that social distancing impacts health conditions [8], especially for persons that are physically vulnerable [9]. This occurs because social distancing affects the maintenance of other essential health services [10, 11].

Children at risk for developmental delay might be impacted by social isolation since they usually require the use of health services. Infants at risk for developmental delay are those who have some intrinsic biological risk or risks associated with the mother [12, 13], as prematurity, low birth weight, hypoxemia, use of ventilatory support, cardiorespiratory resuscitation, and prolonged hospitalization [12–14]. These infants usually present delayed motor, cognitive, and behavioral development, besides limitations on functional activities and restricted participation [15].

Due to social isolation, some therapeutic services provided to these children may be limited. Tele-care seems to be an economical way to expand access to infants at risk to health care during social isolation [16]. Tele-care has the advantage of enabling the performance of functional activities by the infants in the home environment, as well as involvement of the family in the rehabilitation process [16]. In addition, tele-care could be inexpensive for patients and institutions, help prevent the spread of the virus, and provide care for these infants.

Some home-based and family-centered interventions have demonstrated positive results in the cognitive and motor development of infants at risk [17–19]. These interventions usually involve the stimulation using functional activities within the child’s home environment, facilitation of caregiver and infant interaction, enrichment of the environment, and optimizes the inclusion of parents in therapy [20]. Studies have shown that family-centered interventions increase the involvement of the parents in setting goals, and the perception of more favorable environments for development [12, 21].

### STEP protocol

This paper describes a tele-care protocol where parents are coached in how to help their child learn. This STEP (specific task- environment- participation) protocol consists of task and context-based activities and enrichment of the environment, using a family-centered approach for infants at high risk for developmental delay. While some protocols of early intervention programs are published [17], we did not find a family-centered intervention protocol that aims to improve functioning (motor capacity, participation) and optimize environmental facilitators (parent-child interaction and physical home environment) in this population. This type of rehabilitation is based on the biopsychosocial approach and, therefore, might improve the health condition and the quality of life of these children [22].

**Fig. 1:**
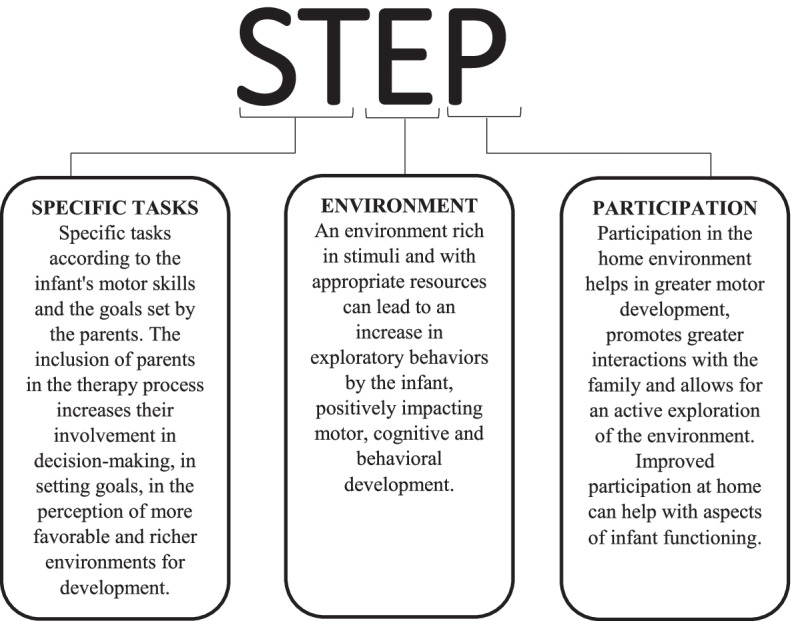
Main principles of the STEP protocol

With the application of the STEP protocol, we want to verify which “ingredients” can be successfully applied in a remote intervention and assessment model. This study aims to investigate whether the STEP protocol, offered remotely, can modify the functioning of infants at risk for developmental delay in the first year of life. The main principles of the STEP protocol are shown in Fig. 1.

It is important that the feasibility of online assessment and intervention protocols be studied. More than knowing if telecare works, we must ask ourselves what aspects of this model in question are viable, functional and effective for the population in question, for example dose and type of intervention.

## Objectives and hypotheses

The primary aim of the study is to compare the motor capacity of infants at risk for developmental delay receiving two types of telecare programs. The secondary objectives are to compare the following aspects between the two tele-care programs: a) compare frequency and involvement components of participation at home; b) compare parent-child interaction in aspects of parental behavior, child behavior and dyad of the relationship between; c) compare the availability of toys and affordances in the physical home environment.

We hypothesize that:

1. Infants who receive the the STEP protocol will have higher scores on motor assessments than those who receive the control intervention;
2. Infants who receive the STEP protocol will have higher scores on participation measures compared to the control intervention;
3. Infants who receive the STEP protocol will have higher scores on parent-interaction and home environment measures compared to the control intervention;

## Methods

This study is in accordance with the Declaration of Helsinki. All ethical approvals were obtained by the Ethics Committee of the University of São Carlos (CAAE number 31256620.5.0000.5504). An online consent form will be given to parents, containing an explanation of the study’s assessments, interventions and methodology. The infant will only be included after the caregivers have signed this form.

### Study design

This is a randomized, controlled, parallel, single-blind clinical study. The allocation rate will be 1: 1. The clinical study protocol was based on the Standard Protocol Items: Recommendations for Intervention Trials - SPIRIT guide [23]. This study was registered in the “REBEC” (Brazilian database for the registration of clinical trials) (registration number: RBR-8xrzjs - Available in http://www.ensaiosclinicos.gov.br/rg/RBR-8xrzjs/ - Issue date: 15 Aug 2021; Protocol amendment number: 02) and will follow the standards established by CONSORT [24].

### Study location

The study will be carried out remotely (online) in its entirety. This factor allows infants from any region of Brazil to be invited to participate. Follow-up clinics for at-risk infants will be invited to publicize the research project.

### Sample size

The study will have a non-probabilistic convenience sample. The sample calculation was performed a priori using the G*Power software. Sample size was determined based on the data obtained in the study by Hielkema [25], from the total score of the Infant Motor Profile (IMP) scale. An effect size of 0.23, statistical power of the test of 90% and statistical significance of 5% were considered. The sample calculation resulted in 52 participants. To guarantee a sufficient number of participants, the inclusion of 15% more participants was established in order to meet a possible dropout rate. Thus, the allocation of 30 participants in each group was determined.

### Recruitment

**Fig. 2:**
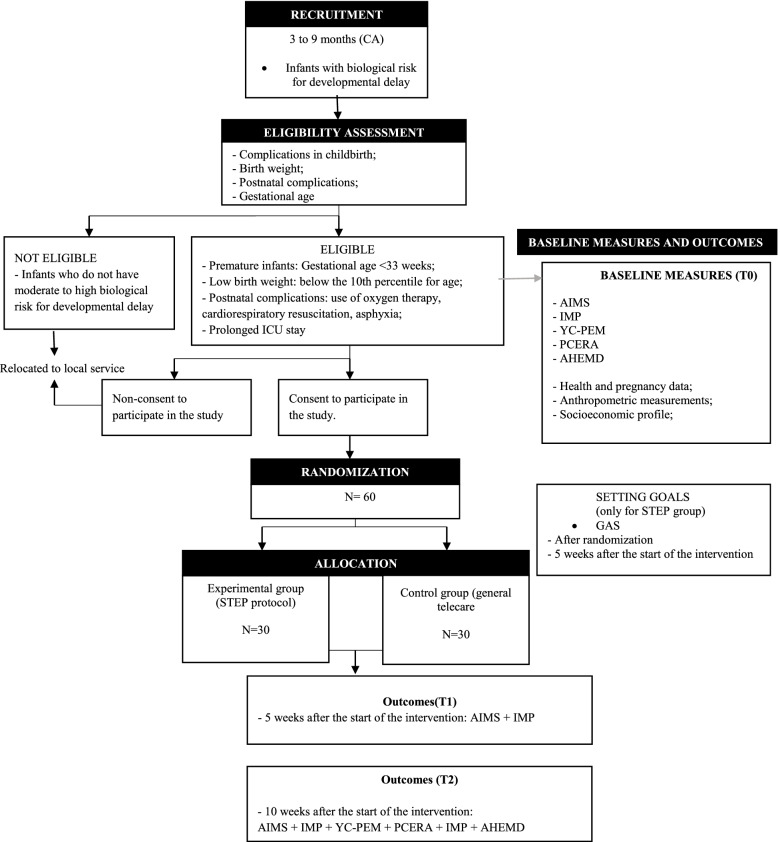
CONSORT flowchart for study participants. Legend: CA - corrected age; PCERA - Parent-Child Early Relational Assessment; IMP - Infant Motor Profile; YC-PEM - Young Children’s Participation and Environment Measure; AIMS - Alberta Infant Motor Scale; AHEMD - Affordances in the Home Environment for Motor Development; GAS - Goal Attainment Scale

The recruitment of participants will take place in two ways: 1) dissemination on social media, radio and e-mail; and 2) contact with professionals at clinics monitoring high-risk infants. In both cases, the initial contact with the parents will be made through an online questionnaire, to verify the initial inclusion criteria. The experimental design, including time points and outcome measures, is represented in the CONSORT flowchart [26] (Fig. 2).

### Participants

Infants from 3 to 9 months corrected age, of both sexes, and with moderate to high risk for developmental delay will be included in the study. Infants who present at least two of the following biological risks for developmental delay will be included: a) prematurity (< 33 weeks of gestational age); b) low weight; c) asphyxia (Apgar score from 0 to 3 for more than 5 min and / or neurological manifestations in the neonate such as seizures, coma or hypotonia); d) the need for cardiorespiratory resuscitation or the use of oxygen therapy; e) prolonged length of stay in the Intensive Care Unit (more than 7 days); f) Abnormal General Movements, assessed by General Movement Assessment (GMA). Infants with the absent fidgety or abnormal fidgety general movements will be included in the study [27]. The infant will be filmed for 5 min, in the supine position, wearing only a diaper. For infants assessed before completing 3 months of corrected age, writhing movements will be assessed. Infants who have cramped-synchronized movements or poor repertoire will be included [28].

Infants will be excluded from the study if they have: a) primary diseases already diagnosed and congenital diseases; b) suspected or confirmed blindness or deafness; c) unstable health status, such as severe respiratory illnesses and frequent or uncontrolled epilepsy.

The caregivers of the infants will also be participants of the study, since they will apply the home intervention. The caregiver that will be responsible for the intervention at home will be the person that is available to apply the intervention in the specified time and that spends most time with the infant. Thus, participants will also be excluded if they have caregivers/parents with the following characteristics: a) cognitive or motor impairments that prevent the understanding or performance of activities administered at home; b) not fluent in Brazilian Portuguese.

Infants may discontinue their participation in the study in the following situations: a) parental withdrawal; b) not performing intervention procedures in the first 7 days of treatment; c) inability to receive online monitoring and evaluation; d) do not perform at least 75% of the planned therapy sessions at home (controlled at the end of the intervention) or do not perform the interventions for two consecutive weeks (controlled weekly).

### Randomization

Randomization of participants will take place after parental consent and first assessments. The infants will be randomized into two groups: 1) control group (conventional guidelines) and 2) experimental group (STEP protocol, using an electronic allocation system (www.sealedenvelope.com). The person responsible for generating and controlling the randomization list will be the only person with access to that list, and will not participate in any other part of the study. The list will be stored on the randomization website and can only be consulted by password.

Children will be allocated to the group by randomization in blocks of 6, with a 1: 1 allocation rate. A person responsible for the randomization list will inform the person responsible for the intervention the assigned group, through a sealed envelope containing the group’s information.

### Blinding

Therapists responsible for conducting assessments will be blinded to group allocation. The persons that will score the assessments are not the same as those that conduct the assessments or the intervention. They will be blind to group allocation by analysing coded videos. Also, the person responsible for data and statistics analysis will be blind. For methodological reasons and due to the nature of the interventions, it will not be possible to blind the therapists that will perform the interventions.

The allocation will not be disclosed to the patient/parents, the therapist from the other group, the evaluators and other research participants.

### Intervention

Therapists responsible for the application of the experimental STEP protocol will receive standardized training consisting of: a) strategies to determine the best activities to be included in the intervention; b) improving and enriching the environment, c) how to guide parents to encourage infant participation at home, with family and in the community; f) components of interaction and how to improve the parent-child interaction. Each group will have its own therapist, and the therapists will not know information about the participants and the progress of the other group. Therapists providing control group intervention will not be aware of the content of the experimental intervention and will receive standardized training.

In both groups, participants will be free to access additional therapy (in person or online) to that offered by this study. In the case of any additional therapy, data on the type of therapy, frequency and duration will be collected. The total dose of therapies will be added as a covariate in the statistical analysis.

To encourage caregivers to continue the study and carry out all stages of evaluations and interventions, reports on the infant’s development will be delivered to the caregiver, to assist in the knowledge about the child’s development and needs. The main principles and differences between the intervention applied in the two groups are shown in Table 1.

**Table 1:** Main principles and differences of intervention between groups

Intervention		
	Control Group	Step Protocol
**Elements**	• Standard functional tasks performed at home by parents to stimulate motor capacity.	• Goals established with parents, through the GAS scale. • Functional tasks performed at home by parents to stimulate motor capacity; • Stimulation of participation at home; • Stimulation of parent-infant interaction; • Environmental enrichment.
**Materials**	• The therapist responsible for the intervention will be trained and will receive a manual with all the motor activities that can be given to the infant, and how to explain each activity to the parents. • Caregivers will receive a booklet with all the functional activities they should perform with the infant, with instructions on how to perform, how many repetitions, which position and what stimuli should be given to elicit the activity.	• The therapist responsible for the intervention will be trained and will receive a manual with all the motor activities that can be given to the infant, and how to explain each activity to the parents. • Caregivers will receive a booklet with all the functional activities they should perform with the infant. The activities will be customized for each infant in the group, according to the skills presented in the assessment and the goals established with parents by the GAS scale. The booklet will have instructions on how to perform each activity, how many repetitions, which position and what stimuli should be given to elicit the activity. • Caregivers will receive a booklet with the main aspects of participation that should be inserted in the infant’s routine, based on the analysis of the participation of each infant. • Caregivers will receive an illustrative manual on the interaction with the infant, covering the main aspects of an adequate interaction, based on the analysis of the facilitators and barriers to interaction, for each infant. • Caregivers will receive instructions on which toys are suitable for the infant’s age, appropriate to the family’s context
**Where?**	• The intervention will take place in the family’s home environment. Parents will be instructed on how to maintain the proper environment (safe, free from external stimuli, with appropriate toys).	• The intervention will take place in the family’s home environment. Parents will be instructed on how to maintain the proper environment (safe, free from external stimuli, with appropriate toys).
**Who executes it?**	• The infants’ parents will carry out the activities. They will receive online guidance from the therapists on all the details of the intervention. • Parents will have a weekly meeting with the responsible therapist to ask questions, check on the infant’s progress, and ensure that the intervention is being carried out with quality.	• The infants’ parents will carry out the activities. They will receive online guidance from the therapists on all the details of the intervention. • Parents will have a weekly meeting with the responsible therapist to ask questions, check on the infant’s progress, and ensure that the intervention is being carried out with quality. • Parents will define with the therapist the goals to be achieved through the GAS scale.
**Dose**	• Motor activities will be performed 30 min a day, 5 days a week.	• Motor activities will be performed 30 min a day, 5 days a week. • The parent-infant interaction + participation will not be measured, but parents will be encouraged to include these aspects in their daily routine.
**Fidelity**	• Parents will record in a pre-determined worksheet everything that was accomplished in each daily session. Afterwards, the therapist will follow the records weekly to see if the parents are following the guidelines exactly.	• Parents will record in a pre-determined worksheet everything that was accomplished in each daily session. Afterwards, the therapist will follow the records weekly to see if the parents are following the guidelines exactly.

An example of the application of the control group protocol and the STEP protocol for the same infant is presented in Supplementary Table 1.

*Control group - conventional guidelines:* this group will receive guidance for the care and development of the infant in the home environment (carried out by the parents). These guidelines will not be specific to the context or the task, but they will have a general character, such as guidance for positioning the infant, stimulation in different positions and general health care, following the guidelines for early stimulation for children aged 0 to 3 years with delay in neuropsychomotor development [29]. A simplified care diary will be provided to the caregiver, to record what the family has accomplished in terms of stimulation and recommended guidelines. The general guidelines and the care diary will be provided by therapists online.

Parents will be instructed to perform the activities for 30 min a day, 5 times a week, totaling 2:30 h of therapy per week. The distribution of therapy throughout the day will be carried out according to the needs and routine of the parents. Outcomes will be collected 10 weeks after baseline measurements.

*Experimental group – STEP protocol:* infants will practice goal directed task and context specific activities in the home environment (carried out by the parents), with assessment and instructions performed online. The protocol will last 10 weeks.

The home intervention will be performed by the parents. All instructions on carrying out the activities will be performed remotely, using the device chosen by the parents. The home practice will be organized according to 3 central principles: 1) establishment of goals; 2) practice of functional tasks and 3) guidance to parents (participation, parent-child interaction and environmental enrichment).

#### Establishment of goals

Initially, three functional objectives will be established according to the Goal Attainment Scale (GAS) [30]. The GAS consists of a measurement technique to quantify the progress of objectives and goals previously defined in an intervention program. The goals will be formulated according to SMART principles (Specific, Measurable, Achievable, Realistic and with a set Time). For each objective, an expected result will be stipulated, and a range of outcome possibilities above or below the expected will be applied. As the objectives are reached (0, + 1 or + 2 in GAS), there will be the establishment of new functional objectives, always maintaining 3 active objectives for the rehabilitation. The establishment of goals will happen after the evaluation of the basic measures and 5 weeks after the beginning of the interventions.

We emphasize that parents will be the main protagonists in setting goals, and the therapists responsible for the intervention will only direct caregivers according to the results of the baseline measurements. For that, a repertoire of possible functional goals will be developed and presented to parents, for the decision of which fit in the family context and the child’s motor capacity.

#### Functional tasks

The activities will be chosen according to the infant’s abilities, and the goals established together with parents. Thus, each family will receive an individualized intervention. The chosen activities will involve task-oriented training, functional activities according to capacities presented, and principles of motor learning. The repetition of each task, guidance on the choice and use of toys, use of sensory cues, identification of the infant’s attempts to produce active movements and self-regulate will be determined.

Parents will be taught by the therapist responsible for the intervention how to perform each activity with their child. Parents will also receive an online booklet, with a photo of each activity, position to be performed, explanation of how to perform the task, and means to stimulate the activity. After the parents receive the booklet with the activities, the therapist will call the parents (by phone or video call) to ensure that the parents understand how to carry out each activity and answer any questions.

Parents will be instructed to perform the activities for 30 min a day, 5 times a week, totaling 2:30 h of therapy per week. This will be the minimum recommended dose. If parents are able and choose to carry out a higher weekly dose of intervention, the researchers will collect data on how many hours per week the family carries out the guidelines. The distribution of therapy throughout the day will be carried out according to the needs and routine of the parents.

#### Guidance to parents

In addition to guidelines regarding functional activities, caregivers will receive recommendations on three topics: environmental enrichment, parent-child interaction and participation.

- Environmental enrichment: the researcher will advise parents on aspects of enriching the environment and guidance on the importance of contextual factors for the development of the infant, taking into account the socio-cultural conditions of each family. Aspects such as the arrangement of furniture, availability of toys, the presence of stimuli (noise, lighting) will be discussed in order to enrich the environment, optimizing the opportunities for learning
- Parent- child interaction: Parents will receive an easy-to-read document with illustrations on how to increase the quality of interaction with their children. In this document, instructions on tone of voice, types of communication, importance of infant responses and cues, incentive for exploring objects, reciprocity in interaction, physical contact and other topics relevant to interaction will be given.
- Participation: This guidance will be developed according to the context of each family and the score on the YC-PEM scale regarding the child’s frequency and engagement at home. In general, parents will be guided in relation to the importance of including the infant in group activities at home, the importance of interacting with family members, in activities of interest to the child and family. The recommendations will also have the character of the infant’s participation at home, in activities such as mealtimes, hygiene and self-care.

An exact weekly time will not be determined for the completion of the above topics. However, we believe that changes in routine will have a significant impact on the stimulation time (participation, interaction) in addition to the time established for the daily activities mentioned.

Parental guidance will be conducted online and weekly. On this occasion, the therapist will hold a meeting by video transmission or telephone call. In this weekly contact, parents’ doubts, difficulties, perceptions of the infant’s improvement and possible suggestions will also be discussed.

### Delivery and fidelity

Therapists in both groups will receive specific training on how to prepare the intervention, how to guide the parents and how to set the therapy goals.

For both groups, an intervention diary will be given to the caregiver, and must be completed on the days of the intervention at home. The “MY HOME STIMULATION DIARY”, contains items such as date, start and end time of the intervention, the duration of each proposed activity, which toys were used, and if there were any difficulty during the intervention. The diary will be delivered online and reviewed by the therapist weekly. To guarantee the fidelity of the interventions, a check-list will be drawn up with all the points that the parents must carry out during the intervention. Opportunities to discuss these points, and the difficulties of the intervention will be offered during the weekly tele-care sessions.

### Outcomes measures

After acceptance to participate in the study and before randomization, the basic measures and anthropometric data, general health data and socioeconomic aspects of each family / infant will be collected (T0).

All assessments will be conducted online. The study will have at least 4 evaluators. All evaluators are trained physiotherapists, with experience in the field of pediatrics and in the scales applied. Evaluators will be trained on the application, scoring and interpretation of all scales. For all outcomes, the inter-examiner and intra-examiner reliability calculation will be calculated using the Intraclass Correlation Coefficient (ICC). For the analysis of the ICC, the classification recommended by Weir (2005) will be considered: null: 0.00; weak: 0.01 to 0.30; regular: 0.31 to 0.60; strong: 0.61 to 0.90 and excellent: 0.91 to 0.99; and full = 1.00.

**Fig. 3:**
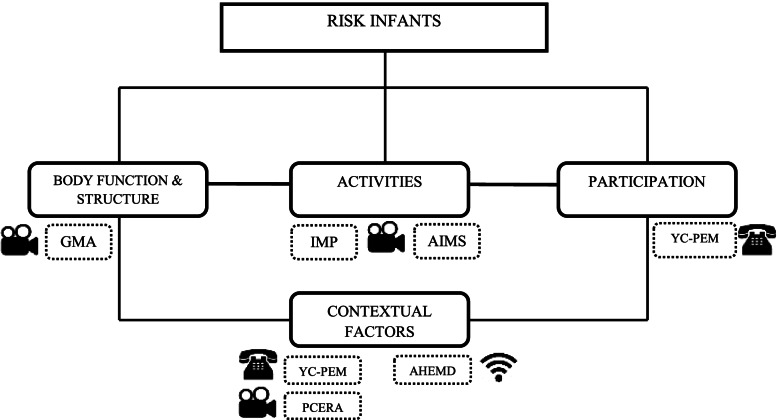
Assessment instruments organized in the CIF scheme. Legend: GMA: General Movements Assessment; PCERA - Parent-Child Early Relational Assessment; IMP - Infant Motor Profile; YC-PEM - Young Children’s Participation and Environment Measure; AIMS - Alberta Infant Motor Scale; AHEMD - Affordances in the Home Environment for Motor Development

The primary outcomes will be motor capacity that will be assessed by the Infant Motor Profile (IMP) and Alberta Infant Motor Scale (AIMS). The secondary outcomes will be: a) participation assessed by the Young Children’s Participation and Environment Measure (YC-PEM), b) the parent-infant interaction assessed by the Parent-Child Early Relational Assessment (PCERA), c) the environmental changes assessed by the Affordances in the Home Environment for Motor Development (AHEMD). The assessment instruments according to the International Classification of Functioning, Disability and Health (ICF) biopsychosocial scheme are shown in Fig. 3.

All these measures will be applied before the intervention (T0). After 5 weeks of intervention (T1) both groups will be evaluated by the AIMS and IMP, to verify the evolution and possibly redirect the activities. After completion of the intervention (T2) all outcomes will be applied again. More details about the outcomes and their applications are found in Table 2.

**Table 2:** Study outcomes

Scale	Outcome	Assessment method	Description	Statistical analysis	Timepoint
Alberta Infant Motor Scale	Primary	Filming with assistance	The scale assesses the motor skills of infants from 0 to 18 months [31]. It is validated for the Brazilian population [32]. Studies demonstrate that home video evaluation of this scale, performed by parents, has validity [33, 34]. We performed the inter-rater reliability analysis with high-risk infants and found values above 0.9, indicating high reliability. The scale consists of 58 items, in which the child is evaluated in the four postures: prone, supine, sitting and standing. In the application of the scale, when the infant is able to perform a certain item, a score of 1 is generated and when he is unable to perform it, a score of 0 is obtained, totaling up to 58 points. The sum of all scales defines the gross AIMS score. This score is transformed into a percentile that represents the child’s motor development [31, 32].	AIMS percentiles	T0, T1 and T2
Infant Motor Profile	Primary	Filming with assistance	Qualitative assessment of spontaneous motor behavior in childhood, with high interobserver reliability (correlation coefficient of 0.95) and strong relationship between their scores and the findings of neurological exams [35]. We performed the inter-rater reliability analysis with high-risk infants and found values above 0.8, indicating high reliability. The IMP was applied because it evaluates beyond the motor milestones, the size of the repertoire, variability and adaptability of the child when performing the movements. The IMP assess infants from 3 to 18 months, or until they have a few months of experience walking independently. The evaluation is recorded for later scoring, and the motor behavior is evaluated in the following postures: supine, prone, sitting, standing and walking. The IMP has 80 items subdivided into five scales: variability-repertoire; variability- ability to select; symmetry; fluency and performance. The scores of each subscale are summed. Higher scores represent greater motor performance.	Weighted average score	T0, T1 and T2
Parent-Child Early Relational Assessment	Secondary	Spontaneous filming	This instrument assesses the early relationship between parents and children aged 0 to 4 years. Through a 5-min film, the scale determines the quality of behavior and the amount of affection in the relationships between parents and children, during the interaction in activities such as food, predetermined tasks, play / free games and separation / meeting between parents and children [36]. It has high reliability in the father (0.86–0.91) and child (0.83–0.87) subscales [36]. The instrument has 65 items subdivided into: parental behavior; child characteristics and dyad of the relationship between them. Higher scores represent a quality of positive interaction.	Total of each subscale: mother / child / dyad	T0 and T2
Young Children’s Participation and Environment	Secondary	Phone call	This is an instrument answered by the caregivers of young children. The YC-PEM presents internal consistency ranged from 0.68 to 0.96 for participation and 0.92 to 0.96 for environment [37]. Although it does not have validation for the Brazilian population, the scale will be used because it jointly assesses participation in activities and the environments that children attend, and is specific for younger children. The YC-PEM assesses the level of activity and participation and the quality of the environment through three sessions: home, daycare/preschool, and community. Higher scores are representative of greater participation and a facilitating environment.	Score of each session regarding frequency and involvement.	T0 and T2
Affordances in the Home Environment for Motor Development	Secondary	Online questionnaire	Evaluation of the quality and quantity of opportunities offered by the family and the environment for the infant’s motor development. The questionnaire is validated, self-explanatory, and is answered by the parents themselves [38]. The scale consists of an initial part, which contains items about the infant and the family (15 items), and three subscales: Indoor and outdoor physical space of the home (10 items), Daily activities (11 items) and Existing toys in the home (20 items) [39]. As it is a self-assessment and self-explanatory questionnaire, the format of this scale allows it to be sent through an electronic form, being self-administered by parents and caregivers, ensuring the reliability of the instrument’s application (Caçola et al., 2011). Scoring is performed by adding the scores for each subscale. Higher scores are representative of greater affordances and opportunities in the home environment. The scale has a maximum score of 49 points [39].	Total sum of the subitems of the scale	T0 and T2

Assessments will take place in four ways, depending on the instrument in question:

1. SPONTANEOUS FILMING: video recording of the infant by parents, on scales that do not require simultaneous instruction. Parents will receive an online document, with all instructions related to the positioning of the infant, in which case the filming should take place, use of toys or stimuli. The PCERA instrument will be applied in this way.
2. FILMING WITH ASSISTANCE: parents will be asked to take part in a video-based telehealth appointment with the evaluator. In the video interaction, the researcher will guide the parents in relation to how to carry out the activities, which positions and stimuli are recommended according to the components of the scale. Parents will apply activities on the infant simultaneously to the video transmission, so that the researcher can correct and guide caregivers in real time. The video stream will be recorded for later scoring. The following instruments will be evaluated in this way: AIMS and IMP.
3. ONLINE QUESTIONNAIRE: the assessment scales that can be self-completed by caregivers will be transformed into online questionnaires. The AHEMD instrument will be evaluated in this way.
4. PHONE CALL: assessments that must be answered by the caregivers but applied by the therapist. The YC-PEM instrument will be evaluated in this way.

### Statistical analysis

The statistical analysis used will follow the standard principles for randomized controlled studies. The SPSS 17 software will be used for data analysis. The analysis of main interest of the study is the comparison between the groups at the end of the intervention. Data from participants who drop out of the study will be stored and all children entered initially (with assessment of the baseline measures) will be included in the intention-to-treat analysis, with a significance level of *p* < 0.05.

To test the difference between the two groups in maternal and infant characteristics (birth weight, gestational age, sex and corrected age) and in baseline measures (AIMS, IMP, YC--PEM, PCERA and AHEMD-IS) will be used the t test for parametric data and the Mann-Whitney test for non-parametric data. In order to verify the effect of the experimental intervention versus the conventional guidelines for primary and secondary outcomes, the t-test and the Mann-Whitney test will be used. The effect size will be calculated using Cohen’s d.

#### Ethics and dissemination

All ethical approvals were obtained by the Research Ethics Committee of the Federal University of São Carlos, and all information collected about the participants will have restricted access, only being disclosed with their consent in unidentifiable formats. The results of this study will be disseminated through scientific articles in international journals and congresses in the area.

#### Data management

Data management will be supervised by the study coordinator. The original files will be stored online and on an external hard drive. Participant data will be stored in a numerical sequence, with no description of the intervention group to which the participant was allocated.

## Discussion

If this study presents favorable results, we can infer that telecare works and is feasible for infants at risk, with the application of interventions by parents. This may be a new look at the therapeutic processes during and even after the pandemic period, in view of the possibility of greater access to health guidelines by people from difficult to reach locations, cost reduction and possible greater frequency of follow-up. Thus, public health policy strategies can be adopted in order to encourage these caregivers to be effectively engaged in the intervention proposals of these children.

## Supplementary Information

**Additional file 1** **: Table S1.**

## Abbreviations

AHEMD-IS: Affordances in the Home Environment for Motor Development
AIMS: Alberta Infant Motor Scale
CONSORT: Consolidated Standards of Reporting Trials
GAS: Goal Attainment Scale
GMA: General Movements Assessment
ICC: Intraclass Correlation Coefficient
ICF: International Classification of Functioning, Disability and Health
IMP: Infant Motor Profile
PCERA: Parent-Child Early Relational Assessment
SMART: Specific, Measurable, Achievable, Realistic, Set Time
STEP: specific task- environment- participation
YC-PEM: Young Children’s Participation & Environment Measure
