# Supplementary material for: Tele-care intervention performed by parents involving specific task- environment- participation (STEP protocol) for infants at risk for developmental delay: protocol of randomized controlled clinical trial
Source: BMC Pediatr. 2022 Jan 20;22:51. doi: 10.1186/s12887-022-03126-3 (PMC8771655; doi:10.1186/s12887-022-03126-3)
Supplement: Supplementary file 1 — Additional file 1 : Table S1. [file 12887_2022_3126_MOESM1_ESM.docx]

An infant with abnormal fidgety meets the study inclusion criteria and caregivers consent to participate. In the motor evaluations, it was identified that the infant sits only with support on the trunk, reaches in the supine position, and supports the trunk with support in the hands in the prone position.

**Table S1:** Hypothetical representation comparing the assessment and intervention procedures of the control group and the STEP group of the same case.

|  | CONTROL | STEP PROTOCOL |
| --- | --- | --- |
| ASSESSMENTS | T0: AIMS, IMP, YC-PEM, AHEMD-IS, PCERA  T1: AIMS, IMP  T2: AIMS, IMP, YC-PEM, AHEMD-IS, PCERA | T0: AIMS, IMP, YC-PEM, AHEMD-IS, PCERA **+ GAS**  T1: AIMS, IMP **+ GAS**  T2: AIMS, IMP, YC-PEM, AHEMD-IS, PCERA |
| GOALS | No goals were set with parents for this group. Standardized intervention. | Goals established together with parents by the Goal Attainment Scale. After meeting with the mother, 3 goals were established:   1. Roll from supine to prone   +2: roll to both sides and return to supine  +1: roll to both sides  0: roll to one side  -1: roll to lateral decubitus  -2: starts movement but does not complete   1. Support of the trunk in prone, for greater interaction with the family   +2: supports the trunk and head with forearm support for 5 minutes  +1: supports the trunk and head with forearm support for 4 minutes  0: supports the trunk and head with forearm support for 3 minutes  -1: supports the trunk and head with forearm support for 2 minutes  -2: supports the trunk and head with forearm support for less than 2 minutes  3) Play in the sitting posture  +2: Play sitting without support for 7 minutes  +1: Play sitting without support for 5 minutes  0: Play sitting without support for 3 minutes  -1: Play sitting without support for 1 minute  -2: Play sitting without support for less than 1 minute |
| FUNCTIONAL ACTIVITIES | Standardized activities for infants of this age, including:  - Reach supine  - Reach in prone  - Sitting stimuli  - Rolling stimulation | Activities established according to the goals identified by the parents and the therapist:  - Rolling stimulation: started in lateral decubitus and started in supine, with the help of the caregiver;  - Support of the trunk in the prone position: visual and sound stimuli for head elevation; reach toys anteriorly and to the side of the body; games and songs with the parents with the baby leaning on the caregiver's trunk.  - Seated reach: Support the trunk as little as possible. High and lateral reach; taking toys out of a basket; anterior reach with toys of different textures. |
| PARTICIPATION | No guidelines are given on participation. | In the evaluation with the YC-PEM at the T0 moment, some gaps in participation were identified, so the parents were instructed in the following aspects:  - Encourage participation when the family receives visits: interacting with other people, playing, babbling.  - Participate with small actions in day-to-day activities, such as bath time, food, and diaper change. |
| INTERACTION | No guidelines are given on interaction. | Guidelines were given in order to encourage the dyad between mother and child:  - Use calm and cheerful tone of voice;  - Demonstrate positive affection, smiling and using expressions of joy;  - Encourage performance through tone of voice, phrases and games;  - Give the infant opportunities to explore the environment and available toys;  - Wait for the infant's response time. |
| ENVIRONMENT | No guidelines are given on the environment. | Parents were instructed on how to make the environment richer according to the contextual characteristics presented in the assessment. Instructions were given on using toys of different textures and sizes and providing safe environments in which the infant can be free and explore. |
